# Supplementary material for: Probing SWATH‐MS as a tool for proteome level quantification in a nonmodel fish
Source: Mol Ecol Resour. 2020 Aug 4;20(6):1647–57. doi: 10.1111/1755-0998.13229 (PMC7689905; doi:10.1111/1755-0998.13229)
Supplement: Supplementary file 2 — Supplementary Material [file MEN-20-1647-s002.pdf]

Supplemental Information for:

Probing SWATH-MS as a tool for proteome level quantification in a non-model fish

Alison A. Monroe, Huoming Zhang, Celia Schunter, and Timothy Ravasi

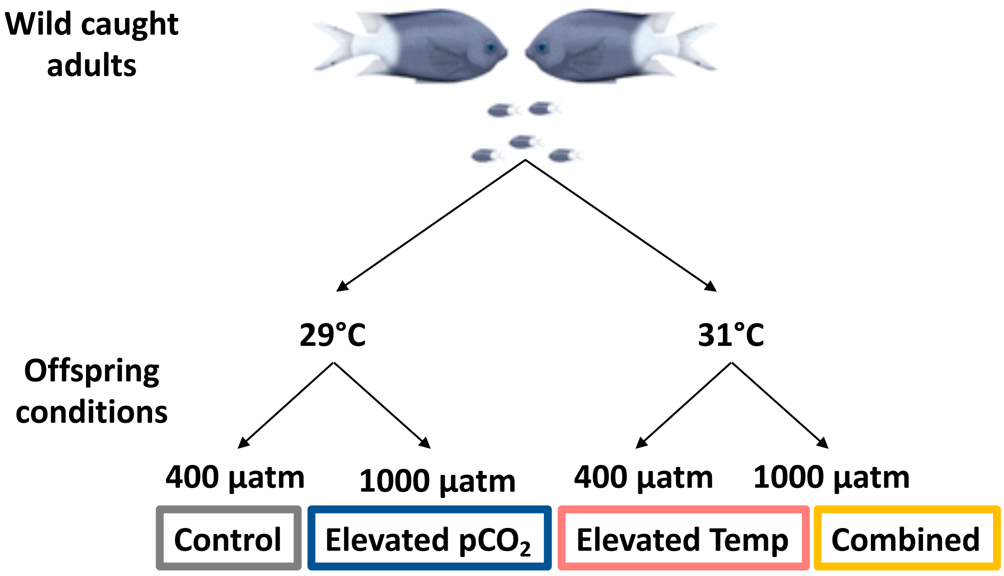

Supplementary Figure 1. Experimental design of samples used in both DDA analysis for spectral library generation and targeted DIA analysis for library validation and differential expression

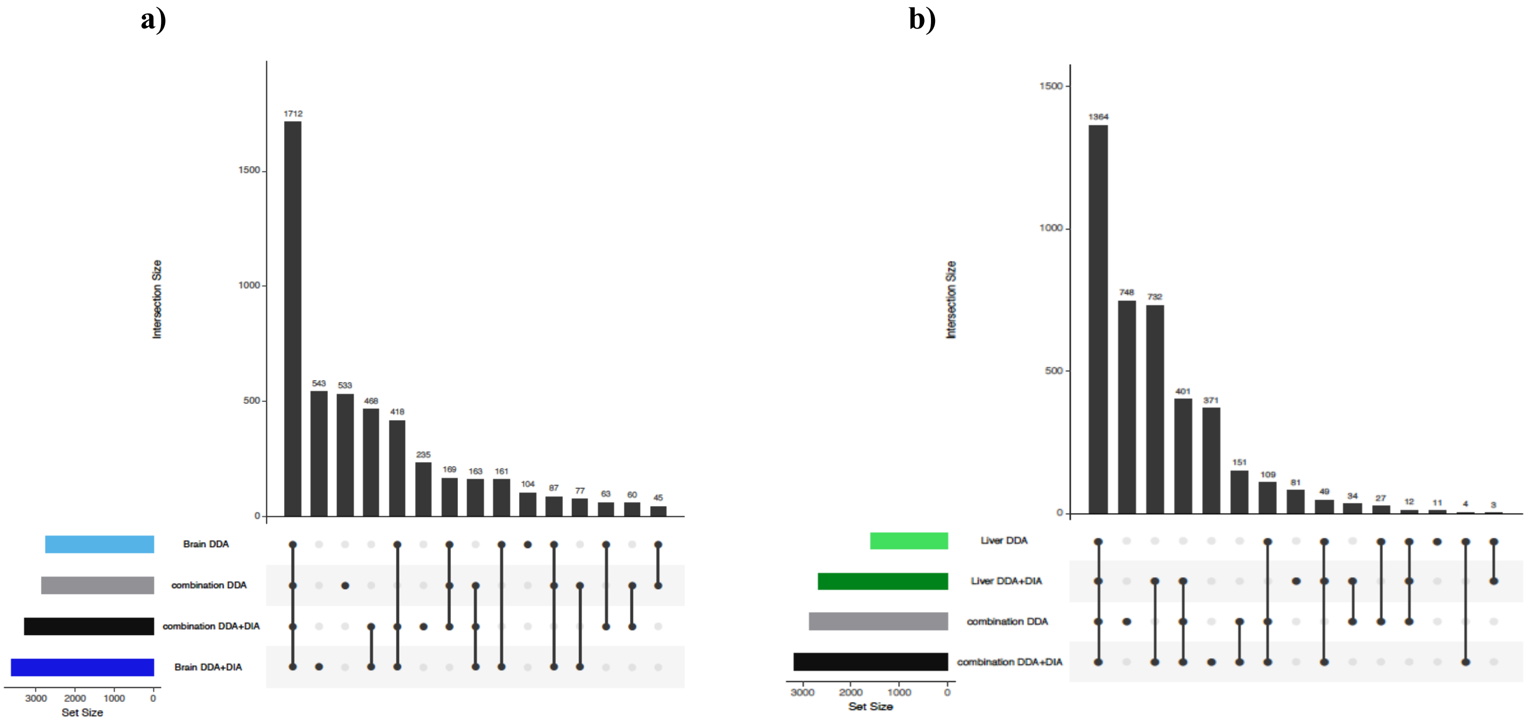

Supplementary Figure 2. Graph showing the overlap of protein groups identified during targeted DIA analysis of a) brain and b) liver tissues against different spectral libraries
